# Illness Representation and Self-Care Ability in Older Adults with Chronic Disease

**DOI:** 10.3390/geriatrics3030045

**Published:** 2018-07-31

**Authors:** Eleanor Rivera, Colleen Corte, Alana Steffen, Holli A. DeVon, Eileen G. Collins, Pamela J. McCabe

**Affiliations:** 1 Department of Health Systems Science, College of Nursing, University of Illinois at Chicago, Chicago, IL 60612, USA; ccorte@uic.edu (C.C.); steffena@uic.edu (A.S.); 2 Department of Biobehavioral Health Science, College of Nursing, University of Illinois at Chicago, Chicago, IL 60612, USA; hdevon1@uic.edu (H.A.D.); ecollins@uic.edu (E.G.C.); 3 Department of Nursing, Mayo Clinic, Rochester, MN 55905, USA; mccabe.pamela2@mayo.edu

**Keywords:** illness representation, chronic disease, heart failure, chronic obstructive pulmonary disease, chronic kidney disease, aged

## Abstract

Chronic illness affects >50% of adults in the United States and accounts for >80% of healthcare spending. The purpose of this study was to determine whether beliefs about one’s chronic disease (illness representation) are associated with self-care activation, emergency department (ED) visits, or hospitalizations. Using a cross-sectional design, we recruited older adults with heart failure, chronic obstructive pulmonary disease (COPD), and chronic kidney disease. The Revised Illness Perception Questionnaire (IPQ-R) measured perceptions about disease. The Patient Activation Measure measured self-care activation. ED visits and hospitalizations were measured by self-report. IPQ-R scores were analyzed using latent profile analysis to identify subgroups. Participants included 187 adults (mean age 65 years, 54% female, 74% Black). We found three subgroups (stable, overwhelmed, and confident). Groups did not differ demographically or by disease. The stable group (few consequences, non-fluctuating pattern) had the fewest hospitalizations. The overwhelmed group (many consequences, fluctuating pattern, high negative emotion) had high hospitalizations and low self-care ability. The confident group (high disease control, well-understood) had the highest self-care ability, but also high hospitalizations. ED visits did not differ by group. We found three subgroups that differ in their illness representation and health outcomes. Findings suggest that assessing patients’ illness representations may have important implications for subgroup-specific interventions.

## 1. Introduction

Chronic disease is one of the most significant health care concerns in the United States. More than 50% of adults have at least one chronic disease, and among adults aged 65 and older, the rate is above 90% [1,2]. Chronic diseases are a substantial source of morbidity and mortality, accounting for 70% of deaths and more than 80% of all health care spending [3]. Currently, an individual’s number of chronic conditions is positively correlated with number of emergency department (ED) visits [4]. Approximately 80% of all inpatient hospital stays are attributed to chronic conditions, and per-person inpatient spending increases with the number of chronic conditions [2]. Moreover, these trends are expected to increase over time [2]. 

Self-care, defined as activities that a person performs to maintain their health, is crucial for chronic disease management [5]. For people with a chronic disease, however, the burden of those activities is much higher [5]. Because chronic diseases are managed rather than cured, the patient is often responsible for carrying out many aspects of the treatment plan. There is evidence that in chronic disease populations, inadequate self-care is associated with more ED visits and hospitalizations [6,7]. As the number of comorbid conditions increases, patients’ self-care decreases [8]. Since most patients will have their chronic disease for an extended period of time, adequate self-care is essential in order to reduce ED visits, hospitalizations, and ultimately, premature death. Unfortunately, the degree of successful self-care varies widely across patients with chronic disease, and intervention efforts to increase self-care have had mixed results [9,10,11,12,13]. 

One reason that self-care varies among patients with chronic disease is that people conceptualize their disease, its treatment, and their role in the treatment plan very differently. Illness representations are one’s beliefs about their illness, including its effect on their life and their treatment [14]. According to illness representation theory, a person’s beliefs about their chronic disease comprise several dimensions, including perceptions of the disease’s symptoms, its cause, its duration, its consequences, and the level of control the individual has over the disease. Studies have shown that different dimensions of the illness representation are associated with self-care related outcomes. For example, one meta-analysis showed a relationship between an individual’s perception of the amount of control they and their treatment had over their disease and their adherence to treatment [15]. Another review showed an association between perceived consequences of the disease and the amount of negative emotion that it caused, as well as the outcomes of anxiety, depression, and quality of life [16]. 

The different dimensions of the illness representation do not exist in isolation. For most studies of illness representation, researchers have examined the relationships between the different dimensions of the illness representation and health care outcomes using traditional analytical methods such as correlation or regression [17,18,19]. However, in this study we want to capture the heterogeneity in total illness representation, rather than focus on each dimension individually. We can accomplish this by using a method of analysis that identifies groups of people within a population who have similar illness representation ‘profiles.’ The advantage of this ‘person-centered’ approach is that rather than assessing the influence of each subscale individually, analysis takes place at the group level, which is based on the illness representation profile as a whole. This would allow researchers to create interventions that can be targeted to each groups’ unique illness representation profile. There have been some studies that have used this strategy to analyze illness representation, such as a recent study in a coronary artery disease population [20], and another of patients with chronic pain [21]. However, none so far have looked at multiple chronic disease populations simultaneously, to see if there are patterns in illness representation profiles that transcend a specific disease. 

The purpose of this study was threefold. First, we conducted a person-centered analysis to identify groups of patients with heart failure (HF), chronic kidney disease (CKD), or chronic obstructive pulmonary disease (COPD) based on their illness representation profiles. Second, we assessed whether the groups differed in demographic and clinical characteristics. Third, we determined whether these groups differed in ED visits in the past year, hospitalizations in the past year, and self-care ability. 

## 2. Materials and Methods 

*Design, Sample, and Setting*. A cross-sectional design was used. Data were collected from March to September, 2017 in the clinics of a large urban academic medical center. A power analysis indicated that a total sample of 180 people would be needed to detect medium effects (*d* = 0.6) with 80% power using analysis of variance (ANOVA) and alpha at 0.05, with the assumption that we would find up to three groups of approximately equal size (60 per group). Effect size was estimated from the average of two studies that examined illness representation using a cluster analysis method, and had an outcome of self-efficacy: one study found an effect size of 0.43 from Pearson’s *r* [22]; the other found an effect size of 0.78 calculated from a t-test [23]. 

A total of 192 patients were recruited from three different types of specialty clinics: heart failure (HF), renal (CKD), and pulmonary (COPD). To select which disease populations we would use for this study, we consulted a list of 20 chronic conditions created by the Department of Health and Human Services Office of the Assistant Secretary of Health [24]. We eliminated from consideration conditions that could have self-report measurement issues (e.g., dementia), conditions with very heterogeneous illness experiences (e.g., cancer), conditions that are often asymptomatic (e.g., osteoporosis), and conditions that are often diagnosed at a young age (e.g., asthma). This left us with HF, CKD, and COPD, which also have similar prevalence rates and extensive self-care treatment components. Patients who were ≥50 years old, could read and understand English, with a diagnosis of HF, CKD (all pre-dialysis), or COPD, without obvious cognitive impairment, who were not living in a nursing home or other care facility, were enrolled. All subjects gave their informed consent before participating in the study. The study was conducted in accordance with the Declaration of Helsinki, and the research protocol was approved by the institutional review board of the University of Illinois at Chicago. 

*Procedures. *The medical records of all patients with an appointment in the clinic on a given day were screened to verify diagnosis (HF, CKD, or COPD) and age. If patients had more than one of the three targeted diseases, they were only approached in the context of the disease for which they had their scheduled appointment. Those meeting eligibility criteria were approached in the waiting room, the study was explained, and interested patients were invited to participate. After consent was obtained, the participant filled out a paper survey document. Upon completion, all participants were given $20 in compensation. A minimum of 60 participants were recruited from each clinic. 

*Measures*. The Revised Illness Perception Questionnaire (IPQ-R) was used to measure illness representation [25]. The IPQ-R uses a five-point Likert-type scale (strongly disagree, disagree, neither agree nor disagree, agree, strongly agree) to assess illness representation along nine dimensions: identity, cause, timeline, consequences, personal control, treatment control, coherence, cyclical timeline, and emotion. Mean scores are computed for each subscale. The description of the subscales is shown in Table 1. Two IPQ-R subscales were not used in this study: the identity subscale (symptoms associated with the disease) and the cause subscale (perceived causes of the disease). Because the causes and symptom profiles of patients with HF, CKD, and COPD differ considerably, the subscales would not necessarily be comparable between these populations. In our study, the participants responded to the same 38 items, with the only change being the name of the chronic disease identified in the questions. 

Reliability and validity testing of the IPQ-R was conducted during initial instrument development (IPQ) and with revisions (IPQ-R) [25,26]. Testing occurred across populations, including post-myocardial infarction (MI), rheumatoid arthritis, diabetes, asthma, and multiple sclerosis [25,26]. Construct validity was demonstrated by the expected pattern of correlations between specific subscales and scores on the Sickness Impact Profile, self-rated health, perceived likelihood of a recurrent myocardial infarction, and health-related distress [25,26]. To evaluate discriminant validity, the instrument authors used the Positive and Negative Affect Schedule; as expected, they found the personal control and treatment control subscales to be positively correlated with positive affect. The emotion and coherence subscales were negatively correlated with positive affect, and positively correlated with negative affect [25]. Cronbach’s alpha scores ranged from 0.79 to 0.89, and six-month test-retest reliability were all 0.5 and above (except for the cyclical subscale, which was 0.35), demonstrating adequate internal consistency and retest reliability [25]. The instrument has been used in dozens of studies since it was developed, including in HF, CKD, and COPD populations [18,27,28]. 

Our IPQ-R Cronbach’s alphas for this sample were: timeline 0.83, consequences 0.73, personal control 0.67, treatment control 0.56, coherence 0.81, cyclical 0.77, and emotion 0.86. The internal consistency of the personal control and treatment control subscales was low. Treatment control in particular is below what is considered acceptable. Removal of any individual items from the personal control or treatment control subscales did not increase the alpha scores. These values, however, are similar to those found in other studies for these populations. In a study of patients with HF, the alpha for personal control was 0.70 and treatment control was 0.55 [27]. One study of patients with CKD had an alpha for personal control of 0.74 and treatment control was 0.46 [28]. Another CKD study found the alpha for personal control to be 0.73 and treatment control to be 0.44 [29].

We examined three outcomes: self-care activation, emergency room visits in the past year, and hospitalizations in the past year. The Patient Activation Measure (PAM) was used to measure self-care confidence, knowledge, and skill, which is referred to as self-care activation [30]. The current 10-item version of the PAM uses a four point Likert-type scale (strongly disagree, disagree, agree, strongly agree). Total scores are computed by summing all scores and transforming them into a standardized 0–100 scale. The score is then categorized into levels: level 1 (≤47.0), level 2 (47.1–55.1), level 3 (55.2–67.0), and level 4 (>67.1). Level 1 (disengaged) indicates a person who is not engaged with and overwhelmed with their disease; level 2 (becoming aware) indicates a person who is still struggling, but has some emerging activation; level 3 (taking action) indicates a person who is comfortable taking action in performing their self-care; and level 4 (maintaining) indicates a person who is maintaining behaviors and taking proactive steps for their health. Previous investigators have also analyzed PAM scores as a dichotomous variable: levels 1 and 2 are considered “low activation”, and levels 3 and 4 are considered “high activation” [31,32]. 

Reliability and validity testing of the PAM occurred during its initial development, as well as during its refinement into a shorter version. Once the initial tool was established, reliability was assessed using Cronbach’s alpha (0.87) and a two-week test-retest (28 of 30 participants had a retest within a 95% confidence interval of the original test) [30]. To test construct validity, the instrument authors found self-care activation to be positively correlated with the SF-8 Health Survey, as well as performance of health behaviors such as healthy diet, regular exercise, and disease-specific treatment adherence [30]. Based on a Rasch analysis, unnecessary items were eliminated, resulting in a shorter version [33]. Since then, the PAM has been used to measure self-care activation in many studies, including in patients with HF, CKD, and COPD [34]. The Cronbach’s alpha for the PAM in our sample was 0.85.

Measurement of ED visits and hospitalizations were based on participant’s self-report of the number of ED visits and hospitalizations in the past year. For ED visits, the question was “In the past year, did you go to the emergency room?” For hospitalizations, the question was “In the past year, have you stayed in the hospital overnight or longer?” Participants indicated yes or no, and wrote in the number of times.

Patient characteristics measured include age (in years), gender, race or ethnicity (African-American/Black, Caucasian/White, Hispanic/Latino, Asian/Pacific Islander, or other), and level of education completed (less than high school, high school, some college, a college degree, or a graduate degree) were measured via self-report. 

We used several variables to characterize the disease severity of the sample. This included self-report of the following: number of years since diagnosis, number of complications (from a list of the four most common complications for each disease) [35,36,37], number of comorbid conditions (from a list of the 10 most common chronic diseases as well as space to write in others) [24], and disease severity (with options of mild, moderate, or severe) [38]. 

*Statistical Analysis. *Descriptive statistics for all variables were computed using STATA version 13 (StataCorp, College Station, TX, USA). Latent profile analysis was performed to identify groups characterized by a unique profile of IPQ-R subscale scores, using Latent Gold version 5.1 (Statistical Innovations, Belmont, MA, USA). Then we assessed differences in outcomes for the various illness representation profiles. Mean scores on the seven IPQ-R subscales were used as continuous indicator variables for the latent profile analysis. We estimated models for up to a five-group (class) solution. We selected the appropriate number of groups based on Bayesian information criterion (BIC) value, residual values, and the theoretical relevance of the solution, which are methods commonly used for this purpose [39]. Once the number and composition of latent profile groups was established, further bivariate analysis and logistic regressions were performed in STATA to assess the relationship between group membership and our outcome variables of patient activation, ED visits, and hospitalizations. 

## 3. Results

### 3.1. Sample Characteristics

A total of 240 patients were approached; 48 declined to participate in the study. One hundred and ninety-two were enrolled (64 with HF, 67 with CKD, 61 with COPD). Five of the participants had a sufficient amount of missing data that not all the IPQ-R scale scores could be calculated; therefore, they were excluded from analysis. The sample (*n* = 187) was comprised of 63 patients with HF, 65 with CKD, and 59 with COPD. The first column of Table 2 shows the sample characteristics. In the total sample, the mean age was 65, 54% of the sample were women, and 74% identified as black. There was a mean of 7 years since diagnosis, an average of 1.7 complications, an average of 3.2 comorbid conditions, and almost half the sample (46%) considered their disease severity to be moderate. All of the outcome variables were skewed, so we dichotomized them as follows: ED visits and hospitalizations were split into either zero visits in the previous year or ≥1 visit; self-care activation was split into either low activation (level 1–2) or high activation (level 3–4). We found a positive relationship between ED visits and self-reported severity (χ^2^(2) = 8.26, *p *= 0.016), and hospitalizations and severity (χ^2^(2) = 11.56, *p *= 0.003). There was also a positive relationship between ED visits and complications (t(185) = −3.097, *p *= 0.0023), and hospitalizations and complications (t(185) = −3.709, *p *= 0.0003). There was a positive relationship between hospitalizations and number of comorbid conditions (t(184) = −2.047, *p *= 0.0421), but no significant relationship with ED visits. We did not find a significant relationship between disease diagnosis, disease years, or self-care activation, and ED visits or hospitalizations (all *p* > 0.29). 

### 3.2. Latent Profile Analysis

**Figure 1:**
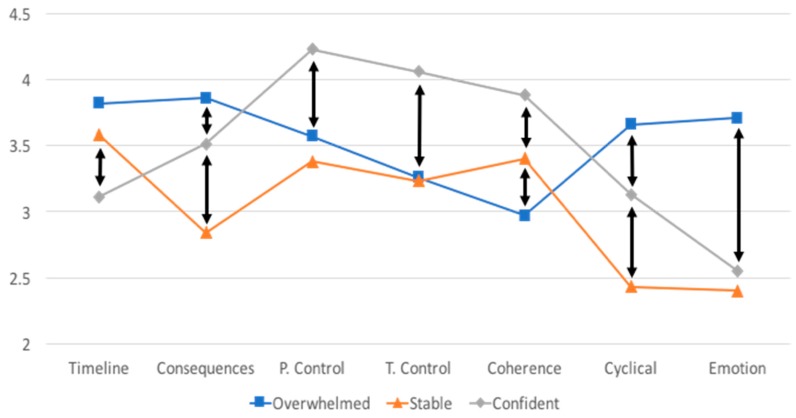
Latent profile analysis results. Arrows indicate statistically significant differences between groups for that subscale. Theoretical range of each subscale is 1–5.

Using the criteria identified above, the latent profile analysis resulted in a three-group solution. The three-group solution had the lowest BIC value (one group = 3155, two groups = 3117, three groups = 3115, four groups = 3117, five groups = 3127). Additionally, for the three-group solution, the residuals showed adequate fit, and the theoretical composition of the solution was sound. Figure 1 depicts the different patterns of illness representation profile scores for the three groups. The first group, which we have labeled “Overwhelmed” (*n *= 80), perceived that their disease had the most consequences, the most fluctuating disease pattern, the most negative emotion, and they had the least coherence (understanding of the disease) of all the groups. The second group, which we have labeled “Stable” (*n *= 61), perceived that their disease had the least consequences and the least fluctuating disease pattern of all the groups. The third group, which we have labeled “Confident” (*n *= 46), perceived that their disease was the least chronic in nature, that they had the most control over their illness, that their illness was most controlled by their treatment, and that they had the most coherent understanding of their disease of all the groups. Given that the latent profile analysis was based on IPQ-R subscale scores, it is not surprising that there were significant differences between the groups for all IPQ-R subscales (Table 2). Tukey post hoc comparisons showed that the majority of the group comparisons differed significantly from each other.

The Overwhelmed group is comprised of nearly equal proportions of patients with HF (36%), COPD (30%), and CKD (34%). The Stable group included more patients with CKD (46%) than HF (26%) and COPD (28%). The Confident group was comprised of generally equal proportions of patients with HF (39%), CKD (33%), and COPD (28%). 

### 3.3. Group Differences

Table 2 shows differences in the three groups for all variables. Group differences were examined with chi-square and ANOVA. There were no significant differences between groups in gender, age, race, education level, or diagnosis. There were significant group differences in number of years with disease, with the Stable group reporting twice as many years with the disease compared to the other two groups. However, the Stable group reported the fewest average number of disease complications compared to the other groups. There were no differences between groups in the number of comorbid conditions or self-reported disease severity. 

Table 2 also shows results for the outcome variables for the three groups. There were no differences between groups in ED visits in the previous year. However, significantly fewer participants in the Stable group were hospitalized in the last year (39.3%) compared with the Overwhelmed group (61.3%) and the Confident group (60.9%) (*p *= 0.020). The differences among the three groups in level of self-care activation did not reach statistical significance (*p *= 0.056), but post-hoc tests of the difference between pairs of groups showed that the Confident group had a significantly greater proportion of high activation scores (level 3 and 4) than the Overwhelmed group (70% vs. 48% respectively, *p *= 0.016). 

Logistic regression analyses were conducted to predict ED visits and hospitalizations (classified as 0 = no visits in the previous year and 1 = one or more visits) and PAM scores (classified as 0 = low self-care activation [levels 1 and 2] and 1 = high self-care activation [levels 3 and 4]) (Table 3). We performed two regressions for each dependent variable—one with the group classifications as variables, and another with group classifications, as well as the two severity variables that had significant group differences (disease years and complications). For ED visits, the unadjusted model containing only group classifications was not significant (*p *= 0.153), but the model was significant when controlling for severity variables (*p *= 0.0270). For hospitalizations, both the unadjusted model and the model with severity variables were significant (*p *= 0.020 and 0.050, respectively). In the unadjusted model, the Overwhelmed group was more than twice as likely to have a hospitalization as the Stable group, but this relationship was attenuated when controlling for severity. For the self-care activation analysis, neither the unadjusted model nor the model with severity variables were significant (*p *= 0.052 and 0.0587 respectively). All regression analyses were free of specification errors or multicollinearity. Likelihood ratio tests demonstrated that the unadjusted model (no severity variables) was more parsimonious for self-care activation, while the full model (including severity variables) was more parsimonious for ED visits and hospitalizations.

## 4. Discussion

The purpose of this study was to identify unique groups of chronic disease patients based on their illness representation profiles, to see if they differed in demographic or clinical characteristics, and to examine the relationship between those groups and three outcomes: emergency room visits, hospitalizations, and self-care activation. Using latent profile analysis, we found three groups with different illness representation profiles: Overwhelmed, Stable, and Confident. These three groups did not differ in their demographic makeup or in the relative proportion of patients with HF, CKD, or COPD, but did have clinically significant differences in outcomes. Our overall IPQ-R scores are consistent with the existing literature for these disease populations [22,40,41], and our overall PAM scores are consistent with the existing literature for these disease populations [31,42,43].

It is somewhat difficult to compare our findings with existing literature because few studies have used similar methods of analysis for illness representation data, particularly with more than one chronic disease population. One study was focused on the relationship between unscheduled care use (urgent care or ED visits) and illness representation profile (using the Brief Illness Perception Questionnaire and a cluster analysis method) in a community population [44]. The authors found that the group that had the highest use of unscheduled care had an illness perception profile with a high degree of consequences, many symptoms, high negative emotion, and low personal control over their illness, which was consistent with our findings [44]. In a study that used a person-centered cluster analysis of illness representation data in a population with hypertension, medication adherence was the worst in a group characterized by high negative emotion, an unstable disease pattern, and low personal control—consistent with our findings [45]. Finally, a recent study was conducted to explore determinants of self-care activation using the Brief Illness Perception Questionnaire and other variables [34]. The sample included patients with HF, CKD, and COPD, as well as type 2 diabetes [34]. Their study used PAM as their clustering variable with illness representation as an outcome variable. Similar to our results, a more positive illness perception (combining all subscales into a single variable) was positively associated with self-care activation in a disease-transcending manner [34]. 

Though the Overwhelmed group had higher scores on the disease severity variables, (disease years, complications, comorbid conditions, and self-reported severity), these scores were either tied for highest with another group or the difference was not statistically significant. They did, however, have the most negative perception of their illness and the worst overall outcomes. The fact that a larger proportion of this group had ED visits and hospitalizations in the past year as well as lower self-care activation, could mean that for this group, perceptions of their disease were more consequential than their perceived disease severity. This is consistent with findings from our review of the literature, which examined studies that used person-centered analysis of the IPQ-R. We found that illness representation profiles characterized by high consequences, an unstable disease pattern, and more negative emotion—profiles that look like the Overwhelmed group—were multifactorial, and included poor coping, lower quality of life, and poor treatment adherence [46]. 

The Stable group had a more positive perception of their illness than the Overwhelmed group. Despite more years with disease, they had fewer disease complications and a smaller proportion reported hospitalizations. Yet the Stable group did not have the highest self-care activation, and though their illness representation pattern was generally positive, they did not perceive that they had a very strong understanding of their disease, or that they had a high degree of control over it. It is possible that the Stable group had the perception that their disease was more out of their control than it actually was. It is also possible that their disease was actually less severe, so they could manage it without a high degree of self-care activation or perception of control over the disease. A 2016 study of COPD patients identified that patients with lower disease severity had higher self-care activation scores [47]. 

The Confident group had, arguably, the most positive illness representation profile, as well as the highest self-care activation. However, hospitalizations in this group were higher than for the Stable group, and at the same level as the Overwhelmed group. This was a surprise, given that the Confident group had the highest perception of control over and understanding of their disease. These findings suggest that this group may have been overly confident in their treatment regimen and their ability to manage their disease. It is possible that the members of this group had a misperception about the course of their illness, or had high self-efficacy, and were hopeful about their disease management in the future. Alternatively, they may have low expectations of how well their disease management could progress. It is also possible that there are other unmeasured variables, such as optimism, that are moderating or mediating these relationships. In a study of patients with Parkinson’s disease, optimism was related to lower perceived disease severity, even when controlling for negative affect [48]. 

Because reliability of the personal control and treatment control subscales was low, group differences based on those subscales should be interpreted with caution. While some studies have found adequate reliability with these two subscales [49,50], other studies have also found low Cronbach’s alpha scores [27,28,29]. It is noteworthy that in the original version of the Illness Perception Questionnaire (IPQ), these two subscales were not separate—they were one control subscale. When the measurement tool was revised (IPQ-R), the control subscale was split into the two subscales: personal control and treatment control. It is possible that the reliability issues seen in these subscales may stem from the decision to separate them into two distinct subscales.

ED visits and hospitalizations were approximately equal for the entire sample, however, only hospitalizations were found to be significantly related to group membership. For the Overwhelmed group, there were similar proportions of ED visits and hospitalizations, which could indicate that each ED visit resulted in a hospitalization. On the other hand, the Stable and Confident groups were similar in the proportion of ED visits, but divergent in hospitalizations. The Stable group had ED visits that did not result in hospitalizations, which could indicate that they were using the ED more proactively, or for subacute care. A study of ED utilization found that older adults with acute conditions were more likely to be admitted to the hospital than those who were presenting with a chronic condition [51]. The Confident group had more hospitalizations than ED visits, which could indicate that they were not seeking out emergent care and were admitted through clinic visits. A previous study of patients admitted with a sepsis diagnosis found that patients who were admitted directly by a health care provider had poorer outcomes than individuals who went to the ED on their own [52]. 

The subgroups found in our study could potentially be interpreted according to “sick role” theory. According to sick role theory, to successfully manage one’s illness, the patient must accept the impact that their illness and its treatment will have on their life [53]. It is possible that individuals in the Stable group—the group with the highest number of years with their disease—had sufficient time to truly accept their sick role, and this is the reason for their better health outcomes. On the other hand, it is possible that what is hindering the Overwhelmed group is their lack of acceptance of their sick role. In future studies, level of acceptance of the sick role could be measured using the Illness Cognitions Scale to see if that can explain some of our group differences [53]. 

Our study had limitations; because it was cross-sectional, we cannot determine if illness representation profiles contribute to outcomes, are a correlate of outcomes, or are a consequence of outcomes. Our disease severity variables and outcome variables were self-reported. While our illness representations were based on current disease perception, our outcomes of ED visits and hospitalizations were retrospective over the last year. The stability of an individual’s illness representation over time has varied in different studies and in different disease populations, with some showing consistency over time [50] and others showing changes [54]. It is possible that ED visits and hospitalizations themselves may have influenced the disease perception of the participants in this study [25]. In addition, our findings from a convenience sample of clinic patients at a single site may not be generalizable to the chronic disease populations in other regions or in other healthcare systems. Our sample consisted of individuals who had access to specialty care, and were actively participating in their care by attending their scheduled visit. We cannot be sure from our results that the illness representation groups identified in this study will apply to other disease populations. 

There are implications of our findings for person-centered care. For example, by screening HF, CKD, or COPD patients to determine their illness representation profile—which could be done quickly by using the 10-item Brief Illness Perception Questionnaire—patients can be identified as Overwhelmed, Stable, or Confident [55]. This assessment could guide choices for different strategies of patient-provider communication or patient education that are targeted to specific illness representation profiles. Appealing to the patient’s specific beliefs about their illness may enhance adherence to treatment. For example, when communicating with individuals in the Overwhelmed group, acknowledging their perception of many consequences, more negative emotion, and an unstable disease pattern may be helpful. Focusing on these factors in the Stable group, however, may not be effective. In terms of future research, the Confident group in particular could be a target for further study, to investigate the root of the mismatch between their disease perception and their disease outcomes. 

## 5. Conclusions

This study was the first person-centered analysis of illness representation to focus on more than one chronic disease. In a clinic sample of 187 patients with HF, CKD, or COPD, we found three distinct groups, each characterized by a unique illness representation profile: the Overwhelmed, Stable, and Confident groups. While the groups were similar in demographic characteristics, they differed in self-care outcomes and incidents of hospitalizations in the past year. Our findings have important implications for assessment and intervention. By assessing a patient’s illness representation, health care providers can tailor patient education, counseling, and care to make them more meaningful to the patient. These clinical goals are consistent with precision health initiatives [56].

## Figures and Tables

**Table 1:** Details of the IPQ-R subscales.

Subscale	Definition	High Score Indicates	Number of Items
Timeline	Whether an individual perceives their disease to be acute or chronic in nature	A more chronic disease perception	6
Consequences	The extent of perceived consequences of the disease	More disease consequences	6
Personal Control	The amount of perceived control a person has over their disease	More control	6
Treatment Control	The amount of perceived control the medical treatments have over the disease	More control	5
Coherence	The perceived level of understanding of their disease	More understanding	5
Cyclical Timeline	Whether an individual perceives their disease to have a stable or unstable pattern from day today	More unstable disease pattern	4
Emotion	Amount of negative emotion an individual attribute to their disease	More negative emotion	6

**Table 2:** Sample characteristics and group comparisons.

Variables	Entire Sample *n *= 187	Groups Derived from Latent Profile Analysis			Chi-Square or ANOVA
		Overwhelmed *n *= 80	Stable *n *= 61	Confident *n *= 46	
Chronic Disease					5.228
HF	63 (33.7%)	29 (36.3%)	16 (26.2%)	18 (39.1%)	
CKD	65 (34.8%)	24 (30.0%)	28 (45.9%)	13 (28.3%)	
COPD	59 (31.6%)	27 (33.8%)	17 (27.9%)	15 (32.6%)	
Age	64.9 (SD = 8.9)	64.5 (8.5)	66.8 (8.8)	63.1 (9.6)	2.468
	Range: 50–88				
Gender					1.704
Female	100 (53.8%)	46 (57.5%)	33 (55.0%)	21 (45.7%)	
Male	86 (46.2%)	34 (42.5%)	27 (45.0%)	25 (54.3%)	
Race/ethnicity					9.201
Black	137 (73.7%)	53 (66.3%)	45 (75.0%)	39 (84.8%)	
Hispanic	15 (8.1%)	7 (8.8%)	5 (8.3%)	3 (6.5%)	
White	30 (16.1%)	16 (20.0%)	10 (16.7%)	4 (8.7%)	
Asian	4 (2.2%)	4 (5.0%)	0 (0.0%)	0 (0.0%)	
Education					0.798
≤High school	96 (51.9%)	38 (48.1%)	33 (55.0%)	25 (54.3%)	
>High school	89 (48.1%)	41 (51.9%)	27 (45.0%)	21 (45.7%)	
**Severity variables**					
Years with disease	6.9 (SD = 8.6)	5.3 (5.2) ^a^	10.3 (12.0) ^b^	5.3 (6.9) ^a^	6.550 **
	Range: 1–55				
Complications	1.68 (SD = 1.0)	1.84 (1.0) ^a^	1.38 (1.0) ^b^	1.80 (1.1) ^ab^	3.965 *
	Range: 0–4				
Comorbid conditions	3.2 (SD = 1.7)				1.32
	Range: 0–8	3.3 (1.8)	2.9 (1.4)	3.3 (1.6)	
Severity					8.713
Mild	56 (31.3%)	19 (24.7%)	24 (42.1%)	13 (28.9%)	
Moderate	82 (45.8%)	34 (44.2%)	23 (40.4%)	25 (55.6%)	
Severe	41 (22.9%)	24 (31.2%)	10 (17.5%)	7 (15.6%)	
**Illness representation variables**					
Timeline	3.57 (SD = 0.87)	3.82 (0.76) ^a^	3.58 (0.81) ^a^	3.11 (0.98) ^b^	10.750 ***
	Range: 1.2–5				
Consequences	3.44 (SD = 0.76)	3.86 (0.61) ^a^	2.84 (0.60) ^b^	3.51 (0.65) ^c^	48.49 ***
	Range: 1.2–5				
Personal Control	3.67 (SD = 0.65)	3.57 (0.57) ^a^	3.38 (0.62) ^a^	4.23 (0.47) ^b^	32.49 ***
	Range: 2–5				
Treatment Control	3.45 (SD = 0.64)	3.26 (0.58) ^a^	3.23 (0.54) ^a^	4.06 (0.45) ^b^	39.12 ***
	Range: 1.8–5				
Coherence	3.33 (SD = 0.85)	2.97 (0.85) ^a^	3.40 (0.77) ^b^	3.88 (0.61) ^c^	20.80 ***
	Range: 1–5				
Cyclical	3.13 (SD = 0.87)	3.66 (0.67) ^a^	2.43 (0.63) ^b^	3.13 (0.80) ^c^	54.37 ***
	Range: 1–5				
Emotion	3.00 (SD = 0.93)	3.71 (0.72) ^a^	2.40 (0.62) ^b^	2.55 (0.74) ^b^	73.75 ***
	Range: 1–5				
**Outcome variables**					
ED visits					3.976
0	83 (44.4%)	29 (36.3%)	32 (52.5%)	22 (47.8%)	
≥1	104 (55.6%)	51 (63.8%)	29 (47.5%)	24 (52.2%)	
Hospitalizations					7.842 *
0	86 (46.0%)	31 (38.8%)	37 (60.7%)	18 (39.1%)	
≥1	101 (54.0%)	49 (61.3%) ^a^	24 (39.3%) ^b^	28 (60.9%) ^a^	
PAM score					5.783
Low activation	84 (44.9%)	42 (52.5%)	28 (45.9%)	14 (30.4%)	
High activation	103 (55.1%)	38 (47.5%) ^a^	33 (54.1%) ^ab^	32 (69.6%) ^b^	

Note: * *p* < 0.05, ** *p* < 0.01, *** *p* < 0.0001. For post-hoc results, different superscripts (a, b, and c) indicate statistically significant differences between groups at *p* < 0.05 level; same subscripts indicate no differences between groups. SD is standard deviation; ED is emergency department; PAM is Patient Activation Measure; HF is heart failure; CKD is chronic kidney disease; COPD is chronic obstructive pulmonary disease.

**Table 3:** Logistic regression analysis.

Variables	ED Visits (≥1)			Hospitalizations (≥1)			Self-Care Activation (High)		
	OR	Z	*p*	OR	Z	*p*	OR	Z	*p*
**Model 1: Groups only (*n *= 187)**									
Stable = 0 Overwhelmed = 1	1.942	−1.92	0.055	2.439	−2.56	0.011	0.768	0.78	0.438
Confident = 0 Overwhelmed = 1	1.613	−1.27	0.204	1.016	−0.04	0.966	0.387	2.37	0.018
Stable = 0 Confident = 1	1.204	0.47	0.635	2.437	2.19	0.029	1.939	1.61	0.107
**Model 2: Groups plus disease years and complications (*n *= 171)**									
Stable = 0 Overwhelmed = 1	1.848	−1.56	0.118	1.701	−1.37	0.171	0.938	0.17	0.866
Confident = 0 Overwhelmed = 1	1.730	−1.38	0.169	0.975	−0.06	0.949	0.352	2.52	0.012
Stable = 0 Confident = 1	1.068	0.15	0.879	1.751	1.28	0.200	2.662	2.15	0.031
Years since diagnosis	1.016	0.78	0.437	0.995	−0.27	0.786	1.030	1.41	0.159
Complications	1.460	2.32	0.020	1.524	2.57	0.010	0.915	−0.56	0.573

Note: OR is odds ratio; ED is emergency department.

## References

[1] Ward B.W., Schiller J.S., Goodman R.A. (2014). Multiple chronic conditions among us adults: A 2012 update. Prev. Chronic Dis..

[2] Anderson G. (2010). Chronic Care: Making the Case for Ongoing Care.

[3] National Center for Chronic Disease Prevention and Health Promotion Chronic Disease Overview. https://www.cdc.gov/chronicdisease/overview/index.htm.

[4] Billings J., Raven M.C. (2013). Dispelling an urban legend: Frequent emergency department users have substantial burden of disease. Health Aff. (Millwood).

[5] Kennedy A., Rogers A., Bower P. (2007). Support for self care for patients with chronic disease. BMJ.

[6] Buck H.G., Dickson V.V., Fida R., Riegel B., D’Agostino F., Alvaro R., Vellone E. (2015). Predictors of hospitalization and quality of life in heart failure: A model of comorbidity, self-efficacy and self-care. Int. J. Nurs. Stud..

[7] Wu S.F., Lee M.C., Liang S.Y., Lu Y.Y., Wang T.J., Tung H.H. (2011). Effectiveness of a self-efficacy program for persons with diabetes: A randomized controlled trial. Nurs. Health Sci..

[8] Dickson V.V., Buck H., Riegel B. (2013). Multiple comorbid conditions challenge heart failure self-care by decreasing self-efficacy. Nurs. Res..

[9] Franek J. (2013). Self-management support interventions for persons with chronic disease: An evidence-based analysis. Ont. Health Technol. Assess. Ser..

[10] Jonkman N.H., Schuurmans M.J., Groenwold R.H.H., Hoes A.W., Trappenburg J.C.A. (2016). Identifying components of self-management interventions that improve health-related quality of life in chronically ill patients: Systematic review and meta-regression analysis. Patient Educ. Couns..

[11] Jiang Y., Shorey S., Seah B., Chan W.X., Tam W.W.S., Wang W. (2018). The effectiveness of psychological interventions on self-care, psychological and health outcomes in patients with chronic heart failure—A systematic review and meta-analysis. Int. J. Nurs. Stud..

[12] Jolly K., Majothi S., Sitch A.J., Heneghan N.R., Riley R.D., Moore D.J., Bates E.J., Turner A.M., Bayliss S.E., Price M.J. (2016). Self-management of health care behaviors for copd: A systematic review and meta-analysis. Int. J. Chronic Obstruct. Pulm. Dis..

[13] Lee M.C., Wu S.V., Hsieh N.C., Tsai J.M. (2016). Self-management programs on egfr, depression, and quality of life among patients with chronic kidney disease: A meta-analysis. Asian Nurs. Res. (Korean Soc. Nurs. Sci.).

[14] Leventhal H., Nerenz D.R., Steele D.J., Baum A., Taylor S.E., Singer J.E. (1984). Illness representation and coping with health threats. Social Psychological Aspects of Health.

[15] Brandes K., Mullan B. (2014). Can the common-sense model predict adherence in chronically ill patients? A meta-analysis. Health Psychol. Rev..

[16] Dempster M., Howell D., McCorry N.K. (2015). Illness perceptions and coping in physical health conditions: A meta-analysis. J. Psychosom. Res..

[17] Pickett S., Allen W., Franklin M., Peters R.M. (2014). Illness beliefs in african americans with hypertension. West J. Nurs. Res..

[18] Fischer M., Scharloo M., Abbink J., van’t Hul A., van Ranst D., Rudolphus A., Weinman J., Rabe K., Kaptein A.A. (2010). The dynamics of illness perceptions: Testing assumptions of leventhal’s common-sense model in a pulmonary rehabilitation setting. Br. J. Health Psychol..

[19] Wilski M., Tasiemski T. (2016). Illness perception, treatment beliefs, self-esteem, and self-efficacy as correlates of self-management in multiple sclerosis. Acta Neurol. Scand..

[20] Kunschitz E., Friedrich O., Schoppl C., Maitz J., Sipotz J. (2017). Illness perception patterns in patients with coronary artery disease. Psychol. Health Med..

[21] Frostholm L., Hornemann C., Ornbol E., Fink P., Mehlsen M. (2018). Using illness perceptions to cluster chronic pain patients: Results from a trial on the chronic pain self-management program. Clin. J. Pain.

[22] Harrison S.L., Robertson N., Graham C.D., Williams J., Steiner M.C., Morgan M.D., Singh S.J. (2014). Can we identify patients with different illness schema following an acute exacerbation of copd: A cluster analysis. Respir. Med..

[23] Lopes A.C., Xavier R.F., Ac Pereira A.C., Stelmach R., Fernandes F., Harrison S.L., Carvalho C.R. (2018). Identifying copd patients at risk for worse symptoms, hrqol, and self-efficacy: A cluster analysis. Chronic Illn..

[24] Goodman R.A., Posner S.F., Huang E.S., Parekh A.K., Koh H.K. (2013). Defining and measuring chronic conditions: Imperatives for research, policy, program, and practice. Prev. Chronic Dis..

[25] Moss-Morris R., Weinman J., Petrie K.J., Horne R., Cameron L.D., Buick D. (2002). The revised illness perception questionnaire (IPQ-R). Psychol. Health.

[26] Weinman J., Petrie K.J., Moss-Morris R., Horne R. (1996). The illness perception questionnaire: A new method for assessing the cognitive representation of illness. Psychol. Health.

[27] Cherrington C.C., Lawson T.N., Clark K.B. (2006). Illness representation of patients with systolic heart failure. Prog. Cardiovasc. Nurs..

[28] Fowler C., Baas L.S. (2006). Illness representations in patients with chronic kidney disease on maintenance hemodialysis. Nephrol. Nurs. J..

[29] Chilcot J., Wellsted D., Farrington K. (2010). Illness representations are associated with fluid nonadherence among hemodialysis patients. J. Psychosom. Res..

[30] Hibbard J.H., Stockard J., Mahoney E.R., Tusler M. (2004). Development of the patient activation measure (PAM): Conceptualizing and measuring activation in patients and consumers. Health Serv. Res..

[31] Zimbudzi E., Lo C., Ranasinha S., Fulcher G.R., Jan S., Kerr P.G., Polkinghorne K.R., Russell G., Walker R.G., Zoungas S. (2017). Factors associated with patient activation in an australian population with comorbid diabetes and chronic kidney disease: A cross-sectional study. BMJ Open.

[32] Aung E., Donald M., Williams G.M., Coll J.R., Doi S.A. (2015). Joint influence of patient-assessed chronic illness care and patient activation on glycaemic control in type 2 diabetes. Int. J. Qual Health Care.

[33] Hibbard J.H., Mahoney E.R., Stockard J., Tusler M. (2005). Development and testing of a short form of the patient activation measure. Health Serv. Res..

[34] Bos-Touwen I., Schuurmans M., Monninkhof E.M., Korpershoek Y., Spruit-Bentvelzen L., Ertugrul-van der Graaf I., de Wit N., Trappenburg J. (2015). Patient and disease characteristics associated with activation for self-management in patients with diabetes, chronic obstructive pulmonary disease, chronic heart failure and chronic renal disease: A cross-sectional survey study. PLoS ONE.

[35] Watson R.D., Gibbs C.R., Lip G.Y. (2000). Abc of heart failure. Clinical features and complications. BMJ.

[36] Thomas R., Kanso A., Sedor J.R. (2008). Chronic kidney disease and its complications. Prim. Care.

[37] Simon H., Zieve D. Chronic Obstructive Pulmonary Disease. http://www.umm.edu/health/medical/reports/articles/chronic-obstructive-pulmonary-disease.

[38] Gupta S., Goren A., Phillips A.L., Dangond F., Stewart M. (2014). Self-reported severity among patients with multiple sclerosis in the U.S. And its association with health outcomes. Mult. Scler. Relat. Disord..

[39] Muthen B., Muthen L.K. (2000). Integrating person-centered and variable-centered analyses: Growth mixture modeling with latent trajectory classes. Alcohol. Clin. Exp. Res..

[40] Morgan K., Villiers-Tuthill A., Barker M., McGee H. (2014). The contribution of illness perception to psychological distress in heart failure patients. BMC Psychol..

[41] Meuleman Y., Chilcot J., Dekker F.W., Halbesma N., van Dijk S. (2017). Health-related quality of life trajectories during predialysis care and associated illness perceptions. Health Psychol..

[42] Halding A.G., Grov E.K. (2017). Self-rated health aspects among persons living with chronic obstructive pulmonary disease. Int. J. Chronic Obstruct. Pulmon. Dis..

[43] Shively M.J., Gardetto N.J., Kodiath M.F., Kelly A., Smith T.L., Stepnowsky C., Maynard C., Larson C.B. (2013). Effect of patient activation on self-management in patients with heart failure. J. Cardiovasc. Nurs..

[44] Lowe R., Porter A., Snooks H., Button L., Evans B.A. (2011). The association between illness representation profiles and use of unscheduled urgent and emergency health care services. Br. J. Health Psychol..

[45] Hsiao C.Y., Chang C., Chen C.D. (2012). An investigation on illness perception and adherence among hypertensive patients. Kaohsiung J. Med. Sci..

[46] Rivera E., Corte C. Person-centered analysis of illness representations: A systematic review of the literature. Proceedings of the Midwest Nursing Research Society Conference.

[47] Korpershoek Y., Bos-Touwen I.D., de Man-van Ginkel J.M., Lammers J.W., Schuurmans M.J., Trappenburg J. (2016). Determinants of activation for self-management in patients with copd. Int. J. Chronic Obstruct. Pulmon. Dis..

[48] Shifren K. (1996). Individual differences in the perception of optimism and disease severity: A study among individuals with parkinson’s disease. J. Behav. Med..

[49] Flora P.K., Anderson T.J., Brawley L.R. (2015). Illness perceptions and adherence to exercise therapy in cardiac rehabilitation participants. Rehabil. Psychol..

[50] Skinner T.C., Carey M.E., Cradock S., Dallosso H.M., Daly H., Davies M.J., Doherty Y., Heller S., Khunti K., Oliver L. (2011). Comparison of illness representations dimensions and illness representation clusters in predicting outcomes in the first year following diagnosis of type 2 diabetes: Results from the desmond trial. Psychol. Health.

[51] Fry M., Fitzpatrick L., Considine J., Shaban R.Z., Curtis K. (2018). Emergency department utilisation among older people with acute and/or chronic conditions: A multi-centre retrospective study. Int. Emerg. Nurs..

[52] Powell E.S., Khare R.K., Courtney D.M., Feinglass J. (2012). Lower mortality in sepsis patients admitted through the ed vs direct admission. Am. J. Emerg. Med..

[53] Berk M., Berk L., Dodd S., Jacka F.N., Fitzgerald P.B., de Castella A.R., Filia S., Filia K., Kulkarni J., Jackson H.J. (2012). Psychometric properties of a scale to measure investment in the sick role: The illness cognitions scale. J. Eval. Clin. Pract..

[54] Kaptein A.A., Bijsterbosch J., Scharloo M., Hampson S.E., Kroon H.M., Kloppenburg M. (2010). Using the common sense model of illness perceptions to examine osteoarthritis change: A 6-year longitudinal study. Health Psychol..

[55] Broadbent E., Petrie K.J., Main J., Weinman J. (2006). The brief illness perception questionnaire. J. Psychosom. Res..

[56] National Institutes of Health What Is Precision Medicine?. https://ghr.nlm.nih.gov/primer/precisionmedicine/definition.

